# An Improved Skin Lesion Classification Using a Hybrid Approach with Active Contour Snake Model and Lightweight Attention-Guided Capsule Networks

**DOI:** 10.3390/diagnostics14060636

**Published:** 2024-03-17

**Authors:** Kavita Behara, Ernest Bhero, John Terhile Agee

**Affiliations:** 1 Department of Electrical Engineering, Mangosuthu University of Technology, Durban 4031, South Africa; 2 Discipline of Electrical, Electronic and Computer Engineering, University of KwaZulu-Natal, Durban 4041, South Africa; bhero@ukzn.ac.za (E.B.); ageej@ukzn.ac.za (J.T.A.)

**Keywords:** attention mechanism, capsule network, classification, dynamic routing, segmentation, skin cancer

## Abstract

Skin cancer is a prevalent type of malignancy on a global scale, and the early and accurate diagnosis of this condition is of utmost importance for the survival of patients. The clinical assessment of cutaneous lesions is a crucial aspect of medical practice, although it encounters several obstacles, such as prolonged waiting time and misinterpretation. The intricate nature of skin lesions, coupled with variations in appearance and texture, presents substantial barriers to accurate classification. As such, skilled clinicians often struggle to differentiate benign moles from early malignant tumors in skin images. Although deep learning-based approaches such as convolution neural networks have made significant improvements, their stability and generalization continue to experience difficulties, and their performance in accurately delineating lesion borders, capturing refined spatial connections among features, and using contextual information for classification is suboptimal. To address these limitations, we propose a novel approach for skin lesion classification that combines snake models of active contour (AC) segmentation, ResNet50 for feature extraction, and a capsule network with a fusion of lightweight attention mechanisms to attain the different feature channels and spatial regions within feature maps, enhance the feature discrimination, and improve accuracy. We employed the stochastic gradient descent (SGD) optimization algorithm to optimize the model’s parameters. The proposed model is implemented on publicly available datasets, namely, HAM10000 and ISIC 2020. The experimental results showed that the proposed model achieved an accuracy of 98% and AUC-ROC of 97.3%, showcasing substantial potential in terms of effective model generalization compared to existing state-of-the-art (SOTA) approaches. These results highlight the potential for our approach to reshape automated dermatological diagnosis and provide a helpful tool for medical practitioners.

## 1. Introduction

The epidermis, the outermost layer of skin, is where malignant cells grow and multiply uncontrollably and abnormally to create skin cancer. The leading cause of skin cancer is prolonged direct exposure to ultraviolet sun rays, which causes melanin, a pigment, to be produced in the top layer of the skin [1]. Moreover, a fair complexion, sunburn, a family history of the disease, and a weakened immune system are risk factors that might lead to the development of skin cancer [2,3]. Skin cancer can take various forms, including squamous cell carcinoma, basal cell carcinoma, and melanoma [4], with melanoma being the most severe type in comparison. Melanoma is a less frequent but more dangerous type of skin cancer and can invade surrounding tissue and cause disfigurement or even death if left untreated at an early stage.

The most prevalent form of cancer in the world is skin cancer. According to the World Health Organization (WHO), in 2020, a total of 1.5 million cases of skin malignancies were detected worldwide, with an accompanying report of over 120,000 fatalities attributed to skin cancer [5]. It is becoming more common in many parts of the world and is now one of the top 10 cancers worldwide. In South Africa, skin cancer is a significant public health concern and has one of the highest incidence rates [6]. According to the South African Skin Cancer Foundation, skin cancer affects up to 80% of newly diagnosed cancer cases in South Africa. It affects one in three people throughout their lifetimes [6]. In South Africa, the prevalence of skin cancer is high for several reasons, including the country’s location in the Southern Hemisphere, where there is higher UV exposure, and the large population of fair-skinned individuals of European descent [7]. Other risk factors for skin cancer in South Africa include exposure to sunlight, outdoor occupations, and a lack of sun protection, such as using hats and sunscreen. To address the growing burden of skin cancer in South Africa, public health officials and health organizations have launched several initiatives to increase awareness of the disease and promote sun safety measures [8].

Clinical diagnosis accuracy for skin lesions in typical clinical settings depends on clinician experience and training. Dermatologists and other health-care professionals have accuracy rates of 60% to 90% for skin cancer identification, with better rates for more experienced practitioners [9,10,11]. Even skilled clinicians can misdiagnose or postpone diagnosis, resulting in poor patient outcomes. Lesions that look like benign skin lesions might make melanoma challenging to diagnose. Asymmetry, border, color, diameter, and evolution (ABCDE) criteria, biopsy, and histological investigation are used by most dermatologists [12,13,14,15,16,17]. Manual visualization and segmentation for pattern analysis make these methods time-consuming, expensive, and inaccurate [18]. Photo or visual examination cannot distinguish malignant from benign lesions. Skin biopsy is limited by its invasiveness, pain, and requirement for additional samples in suspected lesions by various procedures. Non-invasive instruments aid clinical diagnosis [19,20]. Non-invasive dermoscopy procedures collect crucial or irregular skin lesion features, remove reflection, and improve visual impression. Automatically detecting skin lesions may be complex due to artifacts, low contrast, skin tone, hairs, veins, and other visual characteristics like melanoma and non-melanoma [21,22]. Thus, computer-assisted methods that consider pigment networks, streaks, spots, globules, and different skin patterns are needed to help doctors diagnose accurately and quickly [23].

### Motivation and Objectives

In recent years, deep learning-based systems have achieved tremendous popularity in medical imaging and classification. Computer-assisted diagnostics improve skin cancer diagnosis by objectively and quantitatively studying skin anomalies [24]. This can help physicians make better decisions, eliminate misdiagnosis and delay, enhance patient outcomes, increase efficiency, and lower costs. Deep learning algorithms have been proven to identify skin cancer with 90% accuracy, equivalent to or better than human doctors [25]. For years, convolutional neural networks (CNNs) have dominated medical image classification and diagnoses. Their capacity to extract and analyze complex image patterns make them ideal for disease detection, anomaly identification, and tissue classification.

However, CNNs have some limitations, such as not being able to represent spatial relationships between the features, sensitivity to noises [26,27] and limitations in generalizing to new data due to downsampling layers of CNN pooling layers, leading to data loss [28,29,30]. In addition, spatial information, and instantiation parameters (such as the position of low-level features to one another, deformation, and texture information) are not transferable in convolutional neural networks [30]. Thus, the above restrictions result in five major problems:

- **Low Contrast:** Low-contrast skin lesions affect lesion localization accuracy. Some existing technologies may occasionally fail to generate exact, clear edges between various regions in the images during segmentation. Some authors have failed to address the preprocessing method, which might lead to image inaccuracy [25].
- **Variations**: Variations in lesion shape and texture can lead to incorrect region segmentation, which then leads to the extraction of irrelevant features [26].
- **Feature Extraction**: Failure to incorporate crucial spatial relationships among characteristics such as incorrect region features, healthy region features, and extra features that are necessary for the classification purpose [27].
- **Time-Consuming**: Certain classification techniques may require a substantial amount of annotated data to operate optimally, which can be costly and time-consuming, especially for less frequent or specialized forms of skin cancer [28].
- **Lack of Interpretability**: Understanding decisions and how to interpret the retrieved features is not always easy. Because there are more extracted features than previous efforts, the final prediction is more challenging.

Skin lesions are incredibly challenging to classify appropriately because of their similarities in size, color, and overall appearance. To address the first problem, the authors used data augmentation and normalization techniques to capture low-contrast skin lesions by eliminating air bubbles, noise, and artifacts [30]. Segmentation is crucial for an accurate analysis of targeted regions, especially when identifying small abnormalities or lesions. Therefore, active contour segmentation in a snake model has been proposed to effectively segment the region of interest and image borders [31]. Active contours are flexible curves that can deform and adapt to the local features of an image and are especially efficient at capturing and enhancing boundaries based on local image properties. Although CNNs are effective at extracting features directly from intensity values, active contours have the ability to utilize additional information, such as gradient information, to detect edges and boundaries. This can be useful in cases where lesions exhibit subtle variations that are not easily captured by intensity alone. The pre-trained model ResNet50 has also been employed to extract the most relevant features from the image and address the overfitting problem [32]. These approaches have been proposed as potential solutions to address the second problem. Lastly, to address the third problem, we proposed a dynamic routing model known as capsule neural networks (CapsNets) by fusing channel and spatial attention mechanisms [33] to highlight the informative regions and improve the accuracy, generalization ability and interpretability of the model for skin lesion recognition and detection. The routing mechanism of the network suppresses the noise and focuses on the most relevant features of the image. Capsule networks can comprehensively record image features, positions, channels, and spatial relationships through neuron “packaging” [34]. Therefore, capsules can identify specific patterns and mitigate the network’s reliance on extensive datasets [34], effectively improving the model’s capacity to address a broader spectrum of pathological assessment demands [35].

The main contribution to this research area is as follows.

- Developing and implement an active contour segmentation technique for accurately localizing skin lesions within images and applying the ResNet50 pre-trained network to extract essential and relevant features of interest from images.
- Proposing a novel approach by integrating a capsule network architecture fused with the convolutional block attention module (CBAM), which includes dynamic routing and layer-based squashing for feature extraction and classification of segmented skin lesions. Stochastic gradient descent (SGD), a gradient-based optimization technique, is used to optimize the model parameters.
- Evaluating the novel approach on a diverse dataset of skin lesion images and comparing its performance against traditional methods and state-of-the-art techniques.

The rest of the article is organized as follows. Section 2 discusses related work; Section 3 describes the proposed research technique, including the protocol, algorithm, mathematical representations, and pseudocode; the proposed method is compared with existing approaches to offer simulated results in Section 4; and finally, the conclusion and future work is presented in Section 5.

## 2. Related Work

With the rising prevalence of skin malignancies, a growing population, and a lack of competent clinical experience and resources, there is a high demand for AI image diagnosis to assist physicians in medicine. Extensive research has been conducted on automated skin cancer diagnosis [36]. Most skin lesion diagnostic studies followed the standard machine learning method, including preprocessing, segmentation, feature extraction and selection, and classification [32]. Researchers have developed computer-aided diagnosis approaches based on deep learning techniques that differentiate between malignant and benign skin lesions using several image modalities, including histopathology, confocal, clinical follow-up, dermoscopy, and expert consensus [32,36,37]. Deep learning algorithms have demonstrated notable achievements in the field of medical imaging, particularly in the realm of skin cancer detection [38]. Traditional automated skin cancer diagnostic methodologies typically involve two primary components: developing handmade features and utilizing machine learning classifiers for classification [39]. A computer-aided design (CAD) system encompasses several crucial stages, including preprocessing initial dermoscopy images, lesion detection by segmentation approaches, extraction of handcrafted features, selection of features, and classification using machine learning classifiers [39].

Due to its excellent feature extraction, researchers use a convolutional neural network (CNN) for skin cancer detection [39]. However, convolutional neural networks (CNNs) require a lot of training data to recognize images with rotational invariance or other transformations accurately and record spatial relationships between features [40]. Reinforcement learning and pre-trained models were used to solve CNN restrictions [41,42]. The approaches failed to improve, leading to capsule networks (Caps Nets) [43]. This technique improved model accuracy to a greater extent than CNN [44]. The authors of [45] used “faster region-oriented convolutional neural networks (RCNNs) and fuzzy k-means cluster (FKM)” to detect cutaneous melanoma. After refining dataset photos to improve visual information and remove noise and illumination, the faster RCNN constructs a feature vector of a predefined length. FKM breaks the image into different-sized and boundary fragments. FKM cannot always accurately define skin lesion image borders. The Faster R-CNN model may overfit if the training dataset is too small or the model is not correctly regularized. The proposed skin cancer detection method separates benign, malignant, and typical carcinoma [46]. After feature extraction, segmentation, and classification, fuzzy C-means clustering image segments is advised. LVP and LBP extract features from segmented images. The fuzzy classifier identifies images using LVP + LBP recovered features. The enhanced “rider optimization algorithm (ROA)” “distance-oriented ROA” is used best to find fuzzy classifier membership function boundaries in this work. FCM performance may decrease with complex or textured graphics. It may not converge or be locked in local minima. It can be challenging to predict the number of clusters in advance.

To classify dermoscopy images with benign or malignant lesions, [47] offered two new hybrid CNN representations through an SVM categorizer at the output layer. The SVM classifier classifies the first and second CNN representations’ concatenated characteristics. The framework’s performance is compared to dermatology labeling. This model outperformed the latest CNN representations on the public ISBI 2016 dataset. A DNN with optimized training and dermoscopy image learning may identify skin cancer [48]. Combining many dermoscopy datasets provides a solid foundation for DL representation training. The recommended framework trains faster on a small dataset utilizing transfer learning and fine-tuning. Data augmentation improves method performance. A total of 58,032 fine-tuned dermoscopy images were used in this education. The highlighted metrics suggest that the DNN network using customized EfficientNetV2-M outperforms recent deep learning-based multiclass classification representations. The deep neural network (DNN) architecture classifies lesions as benign or malignant. Labeled skin lesions in the data set are categorized using these binary classes. Due to several circumstances, including imaging equipment, illumination, and patient movements, medical images, especially dermoscopy images of skin lesions, can be influenced by noise. Identifying and assessing the lesion adequately might be challenging because noise can hide crucial features and produce artifacts.

In recent years, a deep neural network system used transfer learning to extract features from dermoscopy images and a classifier layer to predict class labels. Study [49] recommends DL for exact lesion extraction. Image quality is improved by “enhanced super-resolution generative adversarial networks (ESRGANs)”. ROI is identified from the complete image after segmentation. For image evaluation, CNNs and modified Resnet-50 models classify skin lesions. Seven skin cancer types from the HAM10000 dataset were randomly selected for this study. The recommended CNN-based technique outperformed the preceding analysis with 0.86 accuracy, 0.84 precision, 0.86 recall, and 0.86 F-score. Image processing and ML synthetically diagnose skin cancer [50]. Graphic low-resolution images are employed to recreate high-resolution images or sequences. CNN representation precision improved with deep learning image super-resolution. CNN’s decision-making and learned qualities are challenging to understand. Many retrieved features make the final prediction harder. CNN training requires a lot of labeled data, especially for high accuracy and generalization, which is time-consuming.

The ISR package and DLNs, like ResNet, VGG16, and InceptionV3, can improve low-quality images for computer-assisted skin cancer detection. Skin cancer features like border, color, symmetry, diameter, texture, size, and form can be analyzed using neural networks. These features are used to classify healthy and cancerous skin using image-based data. The authors of [51] propose “teaching–learning-based optimization (TLBO)” and the upgraded extreme learning machine (ELM) algorithm for flexible melanoma diagnosis. ELM is a quick, accurate feed-forward neural network with one hidden layer, and TLBO optimizes system settings for optimal visual output. Combining these methods may improve melanoma detection by classifying skin lesions as benign or malignant [51].

Study [52] proposed an intelligible CNN-based stacked ensemble framework for initial melanoma skin cancer detection. The stacking collaborative structure employs the transfer learning concept to combine many CNN sub-methods that achieve the same categorization task. The last forecasts are generated by a novel kind called a meta-learner, which uses all of the sub-model predictions. The representation is assessed using benign and malignant melanoma images from an open-access dataset. Using an explainable technique, proficient adaptive clarifications generate heat maps that emphasize the areas within melanoma images exhibiting the highest degree of infection manifestation. Dermatologists can, therefore, understandably interpret the model’s decision.

A new deep transfer learning standard for MobileNetV2 melanoma classification is proposed [53]. MobileNetV2, a deep CNN, diagnoses skin lesions as benign or malignant. ISIC 2020 assesses the presentation of deep learning standards. Class imbalance arises when 2% or less of dataset samples are certain. Augmenting data with random elements reduces session inequality. Studies in [54] show that deep learning outperforms cutting-edge DL algorithms’ accuracy and computing power. The proposed system [54] combines robotic “DL with a class attention layer-oriented skin lesion detection and classification (DLCAL_SLDC)” to identify and classify skin lesions. The DLCAL-SLDC technique classifies skin tumors using dermoscopy. A dull razor removes hair; a typical average filter removes noise in image preparation procedures. Dermoscopy images are segmented using Tsallis entropy to locate suspicious lesions. Capsule network, computer aided diagnosis, and an Adagrad optimizer use DLCAL-oriented feature extractors to extract features from segmented lesions. CAL is constructed to link CapsNets for processing and capture class-specific properties for dependency reporting. SSO-based CSAEs are classified last. DLCAL-SLDC is tested on a benchmark ISIC dataset. An imbalanced dataset in this work introduces a novel DL-based skin cancer detector [54].

The author of [55] employed RegNetY-320 deep learning models for skin cancer classification. Data augmentation was used to rectify the data imbalance to equalize the distribution of skin cancer classifications. Skin Cancer MNIST: HAM10000 has seven more skin lesions. RegNetY-320, InceptionV3, and AlexNet are deep learning-based skin cancer classifiers. Hyperparameters were varied in numerous combinations to adapt the suggested structure. RegNetY-320 outperformed InceptionV3 and AlexNet in accuracy, F1 score, and the receiver-operating characteristic (ROC) curve compared to the imbalanced and balanced datasets. The proposed structure outperformed more conservative techniques. They may help diagnose illnesses early, minimize unnecessary biopsies, save lives, and lower medical costs for patients, skin specialists, and doctors.

FixCaps enhanced dermoscopy image categorization in [56]. FixCaps uses a huge in-height presentation kernel, 31 × 31, at the lowest convolution layer instead of the more common 9 × 9. This may give FixCaps more views than CapsNets. Convolution and pooling lose three-dimensional data when the convolutional block attention segment is added. Collection convolution was used to avoid capsule layer underfitting. The system can reduce calculations and enhance detection accuracy compared to other methods. According to the investigational results, FixCaps had a higher accuracy rate than IRv2-SA, which had 96.49% on the HAM10000 dataset. The study aims to determine how the ability of DL models to generate large data networks affects pharmaceutical manufacturing [57].

The researchers discovered that image determination does not diminish sensitivity, specificity, or accuracy when other features are present. Study [58] focuses on how DL-driven recordkeeping systems help doctors discover skin cancer early and how machinery helps doctors provide quality care. While different and effective augmentation approaches are used, training images improve CNN design accuracy, sensitivity, and specificity. This study proposes extracting and learning essential photo demos using MobileNetV3 design to improve skin cancer detection [58]. Next, the features are used to modify the “hunger games search (HGS) oriented on particle swarm optimization (PSO) and dynamic-opposite learning (DOLHGS).” This adaptation uses a novel feature selection method to determine the most crucial element to improve the image’s presentation. PH2 and ISIC-2016, two- and three-classification datasets, were used to evaluate the DOLHGS’s effectiveness. The suggested technique achieves 88.19% accuracy on ISIC-2016 and 96.43% on PH2. According to the testing, the proposed method surpassed other popular algorithms in classification accuracy and ideal skin cancer analysis features.

Paper [59] addresses possible drawbacks and issues with systems for detecting and classifying skin cancer and ML-based implementations. Additionally, they studied five dermatology-related fields using deep learning: skin disorder measurement using smartphones and personal monitoring systems, dermatopathology visual classification of malignancy, and clinical image categorization. By better understanding machine learning and its many applications, dermatologists will be better equipped to identify potential challenges. This study looked at profound learning studies on skin cancer diagnosis to evaluate alternative approaches. This study also laid the foundations for developing an application for diagnosing skin cancer, and it primarily addresses two problems: deep learning-based skin lesion tracking and image segmentation.

This research [60] suggests a DL-oriented skin cancer categorization network (DSCC_Net) oriented on convolutional neural networks and using three widely accessible standard datasets (ISIC 2020, HAM10000, and DermIS). The suggested DSCC_Net is typically connected to six baseline deep networks for skin cancer classification: ResNet-152, Vgg-16, Vgg-19, Inception-V3, EfficientNet-B0, and MobileNet. To correct the minority classifications in this dataset, they employed SMOTE Tomek. Their DSCC_Net model beats baseline techniques, helping dermatologists and health-care practitioners identify skin cancer. The purpose of study [61] was to (i) address a common class imbalance issue brought about by the fact that persons with skin cancer tend to be smaller than people in good physical shape, (ii) analyze typical production to identify better decision-making, and (iii) create an Android application for a comprehensive intelligent health-care plan to produce reliable deep-learning prediction models. The suggested DL approach was assessed for generalization ability and classification accuracy in association with six popular classifiers. Using an updated CNN and the HAM10000 dataset, this research detected seven cases of skin cancer. A skin lesion classification system utilizing explainable artificial intelligence (XAI) was created, and the outcomes were explained using Grad-CAM and Grad-CAM++ techniques. This method aids in physicians’ early skin cancer diagnosis with 82% classification accuracy and 0.47% loss accuracy. This work carefully categorized skin cancer using a two-tier approach [62].

Data augmentation approaches were employed early in the framework to improve image models for practical training. Based on the encouraging results of medical image processing obtained from a medical vision transformer (MVT), they built an MVT-based classification typically utilized for SC in the second layer of the design. The input image is divided by this MVT into many segments, which are then sent to the transformer in a sequentially similar term embedding. The input image is finally classified using the multilayer perceptron. Through tests on the HAM10000 datasets, they discovered that the MVT-based approach outperforms the most recent methods for classifying skin cancer. Deep learning algorithms can recognize melanoma from dermoscopy images [63]. Fuzzy GrabCut-stacked convolutional neural networks (GC-SCNNs) were employed for the imaging experiment. Several publicly available datasets were utilized to extract image features and classify lesions. The recommended model was shown to detect and classify lesion segments more quickly and accurately compared to the performance of current approaches.

A novel and trustworthy feature fusion model for skin cancer identification was proposed [64]. First, the images are cleaned of noise using a Gaussian filter (GF). While LBP was utilized for manual extraction, Inception V3 performed automatic feature extraction. The learning rate was controlled using an Adam optimizer. Malignant and benign skin cancers were categorized using an LSTM network based on fused features. Their system integrated techniques from DL and ML. For skin lesions on Kaggle, they used the DermIS dataset, which has 1000 images, 500 of which are benign and 500 of which are malignant. They tested their feature-fusion approach against DL- and segmentation-based techniques. After cross-validating their model using a thousand Global Skin Image Collection images, they achieved a detection accuracy of 98.4%. Their method works better than other approaches and yields noteworthy outcomes. Table 1 presents the research gaps in the literature reviewed.

The research gaps are identified by analyzing recent available literature and are summarized in Table 1. To address these gaps, we used preprocessing methods to capture the low-contrast features and active contour segmentation to delineate the skin lesion’s borders precisely. The ResNet50 transfer learning network captures the textural feature maps and addresses the vanishing gradient problem. Also, the lightweight attention mechanism is integrated into convolutional blocks of the CapsNets network to identify the spatial relationship among various features. The CapsNets network reduces the overfitting problem using regularizations and dynamic routing, enhancing model generalization performance.

## 3. Methodology

This study proposes a novel approach for skin lesion classification using a lightweight attention capsule neural network mechanism. The methodology for this study is discussed in the subsequent sections below.

### 3.1. Proposed Novel Lightweight Attention Mechanism-Capsule Neural Network Framework

**Figure 1:**
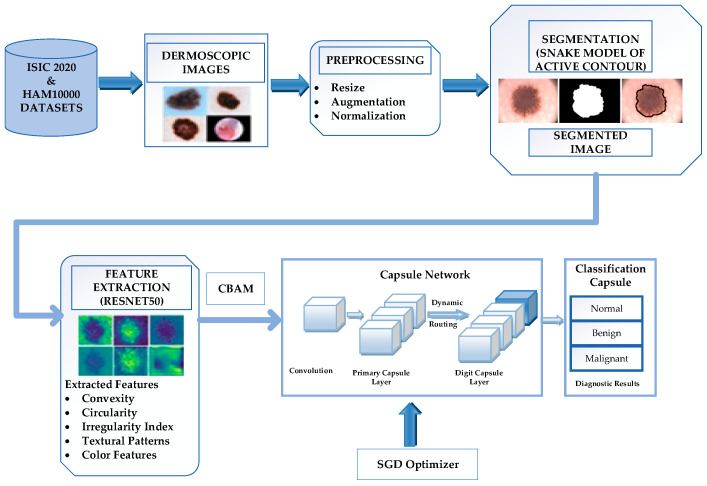
Proposed LA-CapsNet for skin lesion classification.

The proposed framework consists of five distinct phases for skin lesion classification: (1) dataset acquisition and preparation, (2) preprocessing, (3) segmentation, (4) feature extraction and (5) classification, as depicted in Figure 1. Firstly, the datasets were acquired from publicly available domains. The raw images were preprocessed by resizing, normalizing, and augmenting the data, and then the data were divided into training, validation, and test datasets. After augmenting the data, the snake model of the active contour segmentation, which uses a deformable curve to fit the boundaries of an object in an image, is used by removing the irrelevant information from the image. Segmentation helps to focus the learning process on specific features within segmented regions, improving the model’s ability to detect subtle variations and abnormalities. Segmented regions simplify model training and inference and reduce computing complexity. Moreover, segmentation aids in interpreting results by providing a clear delineation of the areas under consideration, which is essential in skin lesion diagnostics. Also, smoothing the image after segmentation can serve as a valid preprocessing step, reducing noise and improving the overall quality of the segmented regions. ResNet50 transfer learning is used for extracting features from segmented images. Apply CBAM, a lightweight attention mechanism, to the feature maps extracted from ResNet. The CBAM module adaptively recalibrates channel-wise and spatial-wise attention to capture essential features in an image. This approach enables the module to accurately capture fine-grained details and direct attention towards relevant spatial regions. After the features have been extracted from the segmented skin lesion image using ResNet50 and CBAM, the features are fused before being fed into the capsule network, improving classification task performance. A guided capsule network is trained on the features extracted by implementing dynamic routing. The stochastic gradient descent (SGD) algorithm is employed to optimize the parameters of a given model. The following section discusses the various phases of skin lesion classification.

### 3.2. Dataset Acquisition and Preparation

The initial step in skin lesion classification is to acquire a high-quality dataset to train our proposed model. Given the need for more high-quality, annotated images of skin lesions, the ISIC datasets are commonly used for automated skin lesion diagnosis. The images were obtained from several centers by diverse operators, utilizing a variety of tools, and stored in multiple formats. The International Skin Imaging Collaboration (ISIC) consortium processed all images, performed privacy and quality screenings, and made the images publicly available in the JPG format. The Creative Commons Attribution-Noncommercial 4.0 International License (CC-BY-NC) governs the use of these databases [65]. Each image includes a specific description of the skin lesion type, verified by competent dermatologists. Various skin lesions are observed in images, including benign and malignant conditions. Benign lesions encompass melanocytic nevus, actinic keratosis, benign keratosis, dermatofibroma, vascular lesions, and lentigo. On the other hand, malignant lesions include melanoma, basal cell carcinoma, and squamous cell carcinoma. Additionally, most of the images are explained by specific information like the anatomical site of the lesion inside the body, as well as the age and gender of the patient.

### 3.3. Image Preprocessing

The primary objective of preprocessing the dataset is to enhance the quality of the unique skin lesion images by removing air bubbles, noise, and artifacts before image capture. The proposed approach involves preprocessing the images through normalization [66], resizing [67], and augmentation techniques. The images’ pixel dimensions were resized to 299 × 299 × 3 to allow effective batch processing and ensure the images aligned with the chosen model architecture. Resizing also helps reduce the model’s computational burden [66,67,68].

#### 3.3.1. Data Augmentation

Data augmentation approaches introduce variation to the dataset by employing image modifications. These modifications include rotations of random angles between −15 to +15 degrees, horizontal and vertical flips applied randomly, adjustments to brightness and random contrast factor between 0.7 and 1.3, randomly cropped regions, and an addition of noise [69]. Data augmentation can enhance the model’s ability to generalize across diverse contrasts, orientations, and other common variations encountered in skin images. Diversity within a dataset can reduce overfitting and improve the model’s efficacy. Consequently, this characteristic is highly advantageous when dealing with datasets exhibiting sparsity [70].

#### 3.3.2. Data Normalization

Normalization, also known as contrast stretching, modifies the range of pixel intensity values [71]. This procedure is a significant preprocessing technique that improves the quality of images by effectively eliminating noise. To normalize the image, it is necessary to modify the intensity of each pixel. Therefore, the image is adjusted to conform to specified values. Each pixel is normalized to maintain the contrast and clarity of the ridge-and-valley pattern [72]. The following is the definition of the normalized image Κ(ρ,υ):

(1) $$Κ\left(\rho ,\upsilon \right)=\left\{\begin{array}{c} N_{0}+\sqrt{\frac{{VAR}_{0}{\left(H\left(\rho ,\upsilon \right)-N\right)}^{2}}{VAR}}\\ N_{0}-\sqrt{\frac{{VAR}_{0}{\left(H\left(\rho ,\upsilon \right)-N\right)}^{2}}{VAR}} \end{array}\right.,ifH\left(\rho ,\upsilon \right)>N$$

where $N_{0}\;$ and ${VAR}_{0}$ are, respectively, the intended (prespecified) mean and variance values, which are defined as follows:

(2) $$\left(H\right)=\frac{1}{M^{2}}\sum_{\rho =0}^{M-1}\sum_{\upsilon =0}^{M-1}H(\;\rho ,\upsilon ),VAR\left(H\right)=\frac{1}{M^{2}}\sum_{\rho =0}^{M-1}\sum_{\upsilon =0}^{M-1}H{\;(\rho ,\upsilon )-N(H))}^{2}$$

To make the calculations that follow easier, we have set $N_{0}=0$ and ${VAR}_{0}=1$ in this work. It ensures that the new pixel intensities for the normalized image will primarily fall between −1 and  1.

### 3.4. Image Segmentation

After preprocessing the images, active contour segmentation is performed to delineate the boundaries of the skin lesions in the images. An active contour, often known as a snake, is a parametric curve that takes the form $Y_{v}=(y\left(v\right),x\left(v\right))$, and it expands or shrinks inside the image such that it can attach itself to the boundary of the item that is being targeted [73]. The development of the snake is modeled using Euler equations, which relate to minimizing the value of certain energy functions. The equations are as follows [74]:

(3) $$bY_{vv}+aY_{vvvv}+M=0$$

where the subscripts signify partial derivatives, b and a are weighting parameters that regulate the snake’s tension and rigidity, and $\;M=(m_{y}{,m}_{x}$) is the equation that describes the relationship between the two variables [75]. Equation (4) derives the entire derivative:

(4) $$M_{s}-t\left(\left|\nabla g\right|\right)\nabla^{2}M-B\left(\left|\nabla g\right|\right)\left(\nabla g-M\right)=0$$

where $\;t\left(\left|\nabla g\right|\right)=e^{-(\frac{\left|\nabla g\right|}{A})}$, $B\left(\left|\nabla g\right|\right)=1-t\left(\left|\nabla g\right|\right),\;\;g=\nabla T_{\sigma }*H$,$\;T_{\sigma }$ is the kernel of the Gaussian distribution, with the standard deviation σ and A as the calibration parameter.

After implementing active contour, the segmented region of interest (ROI) is further processed by creating the binary mask where the lesion region is marked as 1 (white) and the background is marked as 0 (black).

### 3.5. Feature Extraction

Due to its rich, learned feature representation, the segmented ROIs are further processed for feature extraction using the ResNet50 Transfer learning model [75]. The residual building block (RBB) of ResNet-50 is the architecture’s core module. ResNet-50 is a deep convolutional neural network (CNN) architecture known for depth. It is constructed by stacking multiple RBBs and addresses the vanishing gradient problem that can occur in very deep networks. It allows for the training of extremely deep neural networks without the degradation in accuracy that often accompanies increased depth. Therefore, we used a residual neural network (ResNet50) using skip connections to overcome this limitation. The difficulty of the disappearing gradient is alleviated by skipping multiple layers so that the values do not reach the lowest point. The input is added to the layer’s output in a skip connection [76,77,78]. The basic structure of the RBB consists of five convolutional blocks, each taking an input map of a specific size.

Additionally, the architecture utilizes smaller convolutional and max-pooling filters of a specific size across its whole structure. The nonlinear procedure oversees both convolutional layers, each being a convolutional block. Spatial pooling is achieved by utilizing the max-pooling layer, which amalgamates the 2D convolution layers constituting each convolutional block. The rectified linear unit (ReLu), as represented by Equation (5), is designed to address the issue of vanishing gradients. The last component of the system consists of a classifier block comprising an output layer that utilizes a softmax activation function, as well as two fully connected layers with nodes at regular intervals, as described in Equation (6) [79]:

(5) $$g\left(y\right)=\max\left(0,y\right)$$

where y is an input unit.

(6) $$z_{i}=\frac{exp(y_{i})}{\sum_{m=1}^{m}exp(y_{m})},with,\;\;\;\;y_{i}=\sum_{m=1}^{K}I_{m}V_{mi}$$

where $z_{i}\;\mathrm{and}\;y_{i}\left(\left\{i=1\ldots \ldots m\right\}\right)$ is corresponding layers where $I_{m}$ is the initiation of the penultimate layer node, $V_{mi}\;$ is the weight connecting the penultimate layer $y_{i}$ to the softmax layer, K is the number of input nodes, and M is the total number of output nodes (classes). The input units to the softmax layer come after the output.

#### Design of Residual Building Block (RBB) in ResNet50

**Figure 2:**
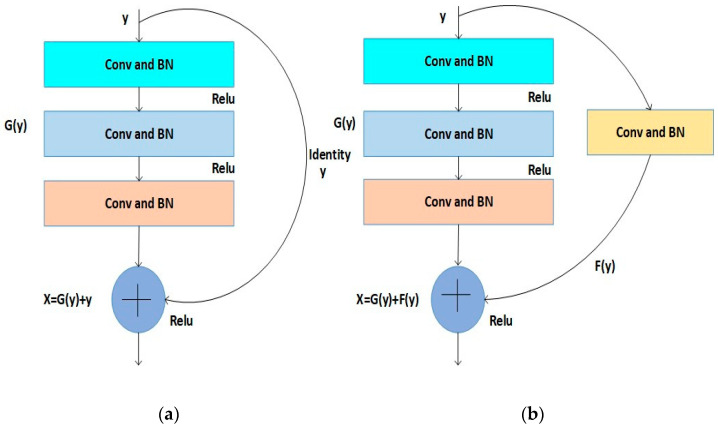
(**a**) RBB-1. (**b**) RBB-2.

The functionality of ResNet-50 is reliant on the presence of the residual building block (RBB). The RBB uses direct connections and bypasses convolutional layer blocks. The vanishing or exploding gradients can be mitigated using these computational techniques to optimize the trainable parameters during error backpropagation. This approach can contribute to developing a more complex CNN architecture and improve the overall effectiveness of defect diagnostics. The ReLu activation function, numerous convolutional layers (Convs), batch normalizations (BNs), and a single shortcut make up the RBB. RBB-1 and RBB-2 are representative of two distinct RBB topologies, as illustrated in Figure 2. Both RBB-1 and RBB-2 have three Conv and BN layers. The identity *X* is the shortcut in RBB-1, as shown in Figure 2a. F is a nonlinear function in RBB-1 for the convolutional path. Equation (7) is used to formulate the output of RBB-1. Figure 2b represents the RBB-2 structure in which RBB1 is substituted as shortcut Conv and BN layers. Equation (8) defines the result of RBB-2, where F represents the shortcut path [76,77,78,79]:

(7) $$x=G\left(y\right)+y$$

(8) $$x=G\left(y\right)+F\left(y\right)$$

Following the initial convolutional layer of ResNet-50, a series of RBB-1 and RBB-2 blocks are sequentially stacked.

**Figure 3:**
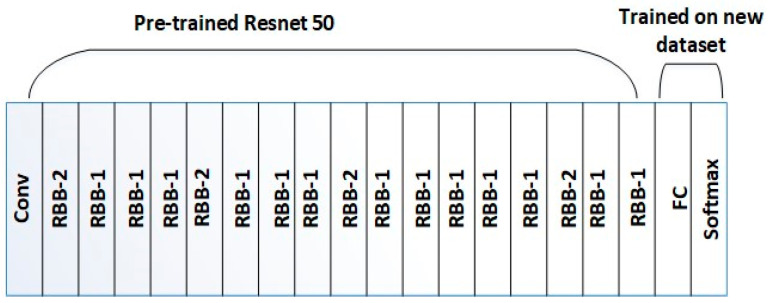
Design of ResNet50.

Figure 3 shows the proposed ResNet-50 structure. The first 49 layers of ResNet-50 are transferred (Figure 3 shows 1+16∗3=49 Conv layers). Next, the softmax classifier and an additional fully connected layer (FC) have been added to the architecture to adapt the class labels of the liability diagnosis dataset to ResNet-50. Improved feature extraction and deeper network layers would enhance the final prediction accuracy of the suggested CNN (ResNet-50) for fault diagnosis. ResNet-50 extracts features from the altered images created by the signal-to-image technique. These characteristics would then undergo fault classification training. The output of the final fully connected layers in the ResNet consists of a 2048-dimensional feature vector for each image. Let $x-g_{ji\;}$ be the feature recovered from ResNet-50 and FResNet the nonlinear function of $RGB{Pixel}_{j}$, i=1…2048. Equation (9) represents the transition process [76,77,78,79].

(9) $$x-g_{ji}=G_{ResNet}(RGB{Pixel}_{j})$$

The features extracted from ResNet50 include convexity, circularity, irregularity index, textural patterns, color features, and region of interest for feature representation and to enhance prediction accuracy.

### 3.6. Skin Lesion Classification Using Lightweight-Guide Capsule Neural Network

The extracted features from ResNet50 are fed into the lightweight mechanism CBAM to focus on the most relevant features for the classification of skin lesions.

#### 3.6.1. Convolutional Block Attention Mechanism (CBAM)

**Figure 4:**
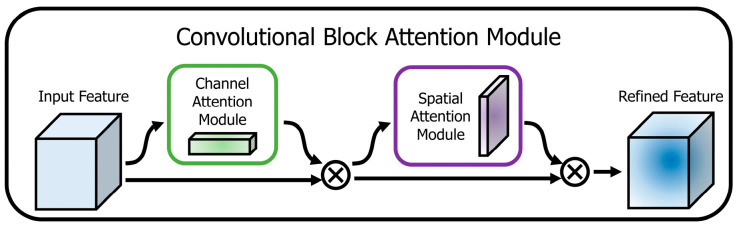
Convolutional block attention mechanism [80].

The CBAM is a lightweight mechanism that is relatively less complex and directs the models to selectively focus on the most prominent characteristics within the feature map. This process reduces irrelevant information and accentuates the regions that provide the most valuable insights for improved classification accuracy and computational efficiency [80]. As shown in Figure 4, it first extracts the channel-wise and spatial-wise attention features, which are then multiplied to highlight informative regions within skin lesion images and improve the accuracy and interpretability of deep learning models for skin cancer detection [80]. By integrating the attention mechanism into the network capsule architecture at the convolution layer level, we can enable the model to focus on relevant regions of the lesion images selectively. Therefore, to increase capsule attention to the object and decrease spatial information loss from convolution and pooling, the feature maps from CBAM are fed into the capsule network [81]. As a result, the network successfully mitigates overfitting without encountering dropouts [82]. The attention process is presented in Equation (10):

(10) $$F^{\prime }=M_{s}(M_{c}(F)⊗F)⊗F$$

The symbol “⊗” represents the operation of element-wise multiplication. Multiplication propagates the attention values in the directions indicated by the operators: values in one channel propagate along the spatial dimension and vice versa [81]. In this context, we refer to channel attention as $Mc(R^{c\times 1\times 1})$ and spatial attention as $Ms(R^{1\times 1\times H\times W})$. The feature map F is the result of the convolutional layer. The refined feature output is denoted by F′.

#### 3.6.2. Development of Proposed Capsule Neural Network

The capsule network (CapsNets) is proposed as a substitute for conventional convolutional neural networks (CNNs) to address challenges that require hierarchical and spatially aware feature extraction. The primary objective of capsule networks is to address the inherent constraints of convolutional neural networks (CNNs) when recognizing intricate spatial hierarchies and retaining pertinent information about the relative placements of features [82].

**Figure 5:**
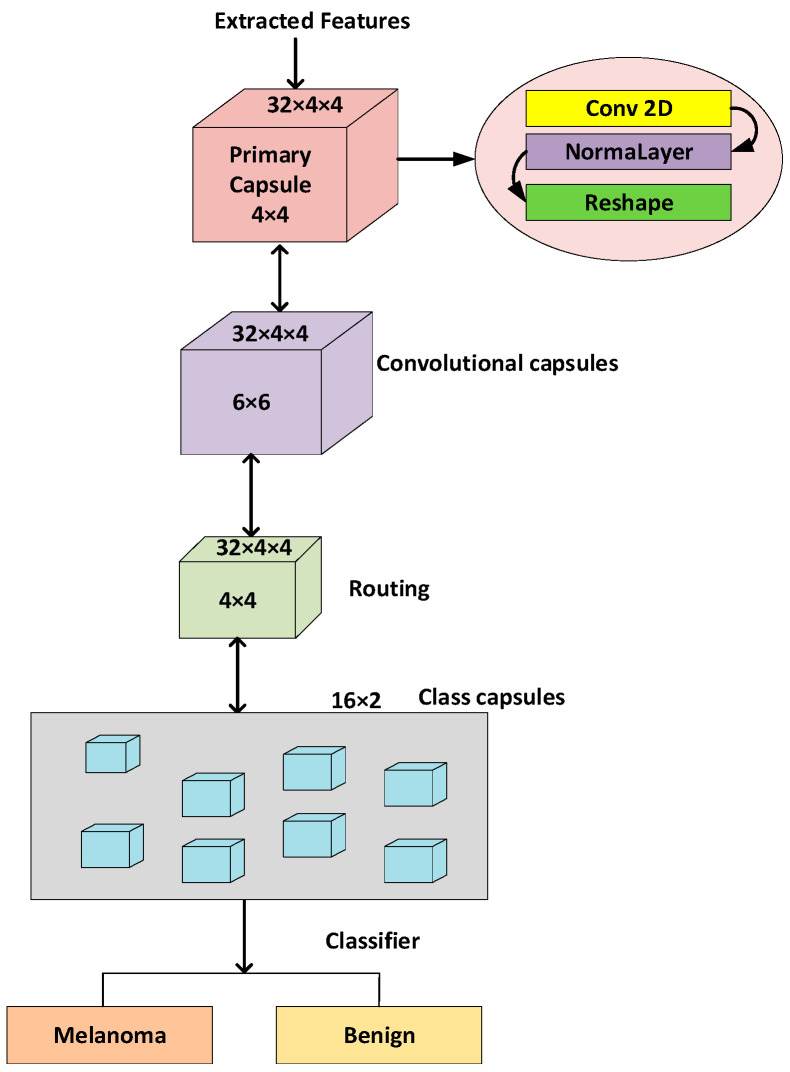
Proposed capsule network model.

A capsule is a cluster of neurons arranged in a vector-like structure responsible for encoding and representing postural data. An object’s activation probability, or simply the possibility that it exists, is reflected in a capsule’s length. Because of this, it is much simpler to derive a part–whole relationship given only the data embedded in one computing unit that represents the portion. Next, the routing algorithm will endeavor to connect each lower-level capsule and a single higher-level capsule at an elevated level that fulfills its criteria [83]. Expectation-maximization (EM) routing is used to determine the posture of each capsule in layer L + 1 based on applying the Gaussian approach to the “minimum description length” principle. This principle allows for the most substantial data compression by selecting the best hypothesis or regularization. The decision to activate the capsule in layer L + 1 is made based on the votes received from layer L. A matrix represents the pose of the capsule, and the EM algorithm is employed to calculate the activation probability [84]. The inverted dot product was utilized in the model, an algorithm for directing the flow of attention that uses a matrix-designed posture within a capsule. As shown in Figure 5, our capsule system consists of one primary capsule, two convolutional capsules, and then dual-class capsules, each dedicated to a specific class. The primary capsule, which generates the initial low-level capsules, receives the extracted features to fulfill its functions. The main capsule will apply one convolutional layer to the retrieved features, and it will standardize the output and then restructure it to construct matrix capsules of size $R^{\sqrt{h_{d}}x\sqrt{h_{d}}}$, where $h_{d}$ denotes the gathered quantity of unknown layers that comprise a capsule [85]. The capsules of the first convolutional layer, also known as the parents, are fed information from the main layer capsules, also known as the children. These capsules then update their parents, and so on. Equations (11) and (12) are used to create the convolutional capsule layers, and each layer has 32 capsules of size 4 × 4, making a total of 64 capsules. To direct a child capsule j located in layer $C(N_{j}^{C})\;\;$ to a parent capsule i located in layer $C+1(N_{i}^{C+1})$, a vote $M_{ji}^{C}\;\;$ is first generated for each child and then applied to each parent by using the weights that have been assigned between the two levels $Z_{j,i}^{C}$. At the beginning, the poses $N_{i}^{C+1}$ of all of the parents are initialized to zero [86].

(11) $$M_{ji}^{C}=Z_{j,i}^{C}\cdot N_{j}^{C}$$

The routing agreement $F_{ji}^{C}$ between each parent and all of their children is determined by applying the dot-product similarity and using the votes $M_{ji}^{C}\;$ that the children have cast.

(12) $$F_{ji}^{C}=N_{i}^{C+1^{L}}\cdot \;M_{ji}^{C}$$

A softmax function is used to calculate the routing coefficient known as $H_{ji}^{C}$. This function is used after the agreement scores are passed through it.

(13) $$H_{ji}^{C}=\exp\left(F_{ji}^{C}\right)/\sum_{i^{\prime }}\exp(F_{ji^{\prime }}^{C})$$

(14) $$N_{i}^{C+1}=\sum_{j}H_{ji}^{C}M_{ji}^{C}$$

A normalization layer is then implemented as a final layer to improve the routing’s convergence.

(15) $$N_{i}^{C+1}=LayerNormal(N_{i}^{C+1})$$

The calculation processes for the capsule layers by the reversed technique are shown by Equations (11)–(15) [87]. The primary iteration is a sequential procedure in which the values of all capsule layers, excluding the initial layer, are calculated. The subsequent iterations occur simultaneously, leading to enhanced performance throughout training. The class capsules make up the last layer of the capsules. The feature vector is heavily compressed in these layers to feed it into a layer for linear classification [88]. The classifier is shared across the class capsules, and this layer is utilized to get the forecast logits. Equations (11)–(15) specify the routing method used to build each of the two class capsules. The size of each class capsule is 16 [89].

#### 3.6.3. Stochastic Gradient Descent (SGD) Optimizer

We optimized model parameters using gradient-based algorithms like stochastic gradient descent (SGD). SGD helps analyze large datasets and complex models. The SGD optimization method trains on labeled data. When used for convex and continuous optimization, stochastic gradient descent (SGD) is conceptually stable [90]. It first claims that minimizing training time reduces generation error. Since the model does not meet identical data instances several times, it cannot use memorization and must build generalization skills. Deterministic gradient descent is a version of SGD without gradient noise. This broadside emphasizes stochastic optimization, but its principles and methods apply to deterministic gradient descent [91]. In this context, we focused on minimizing the cost function.

We utilized gradient-based optimization algorithms, such as stochastic gradient descent (SGD), to optimize the model’s parameters. SGD is very helpful for analyzing massive datasets or intricate models. The SGD optimization algorithm’s goal is to use labeled data to train. The stochastic gradient descent (SGD) algorithm demonstrates conceptual stability when applied to convex and continuous optimization problems [89]. Firstly, it posits that minimizing training time yields the advantage of reducing generalization error. This limitation arises because the model does not encounter identical data instances several times, preventing it from relying on memorization and necessitating the development of generalization capabilities. Deterministic gradient descent is considered a specific instance of stochastic gradient descent (SGD) deprived of gradient noise. The ideas and approaches established in this broadside are also relevant to deterministic gradient descent despite this broadside emphasizing stochastic optimization [90]. In this context, we focused on minimizing the cost function.

(16) $$H\left(\theta \right)=E[L\left(\theta ,v\right)]$$

where θ is a vector of parameters that need to be optimized,v is a random vector, L is a loss utility, and E is a function that takes the expectation over v.

For instance, in the difficulty of classifying data utilizing a neural network, θ signifies the vector that contains all of the tunable weights in the neural network, v = (M, N) is the couple of the feature vector M, and the class label N, and L is a continuously differentiable loss, such as the mean squared error, the cross-entropy loss, or the (multiclass) hinge loss. Learning as θ can be accomplished through the use of gradient descent [92].

(17) $$\theta^{\left[new\right]}=\theta^{\left[old\right]}-\mu Q^{\left(\theta^{\left[old\right]}\right)}$$

where μ>0 is positive step size, then:

(18) $$Q\left(\theta \right)=E\left[\frac{\partial L(\theta ,v)}{\partial \theta }\right]$$

It is possible that evaluating the expectation outlined in (16) is either undesirable or impossible. This expectation is given a close approximation in SGD by using the sample average, which leads to the following update rule for  θ [92]:

(19) $$\hat{Q}\left(\theta \right)=\frac{1}{\varepsilon }\sum_{j=1}^{\varepsilon }\frac{\partial L(\theta ,v_{j})}{\partial \theta }$$

(20) $$\theta^{\left[new\right]}=\theta^{\left[old\right]}-\mu \hat{Q(}\theta^{\left[old\right]})$$

where hat ∧ indicates that the flexible beneath it is being estimated, ε≥1 is the size of the minibatch, and $v_{j}$ marks the $j^{th\;}$ sample, which is normally picked at random from the training data. Since $\hat{Q}\left(\theta \right)$ represents a random vector, it is rewritten as follows [92]:

(21) $$\hat{Q}\left(\theta \right)=Q\left(\theta \right)+E^{\prime }$$

to make the distinction between its deterministic and random components, with the random vector $E^{\prime }$ representing the estimate error that results from substituting the expectation with the sample average.

We examine the subsequent second-order approximation concerning $H\left(\theta \right)$ around a point $\theta_{0}$:

(22) $$H\left(\theta \right)\approx H\left(\theta_{0}\right)+Q_{0}^{T}\left(\theta -\theta_{0}\right)+\frac{1}{2}{\left(\theta -\theta_{0}\right)}^{T}R_{0}\left(\theta -\theta_{0}\right)$$

If the superscript T indicates a transposition, the gradient at 0 is denoted as $Q_{0}=Q(\theta_{0})$, and:

(23) $$R_{0}=\frac{\partial^{2}H\left(\theta \right)}{\partial \theta^{T}\partial \theta }{|}_{\theta =\theta_{0}}$$

is the Hessian matrix at the value $\theta_{0\;}$ position. Take note that the definition of $R_{0}$ calls for it to be symmetric. Regarding the gradients of function $H\left(\theta \right)$ with respect to θ, the variable about $\theta_{0}$ can be assessed using the following approximation:

(24) $$Q\left(\theta \right)\approx Q_{0}+R_{0}(\theta -\theta_{0})$$

(25) $$\left(\theta \right)=Q_{0}+R_{0}(\theta -\theta_{0})+E$$

where E includes the errors in Equations (20) and (22). When Equation (24) is applied, the learning rule (14) transforms into the linear system described below [92]:

(26) $$\theta^{\left[new\right]}=\left(T-\mu R_{0}\right)\theta^{\left[old\right]}-\mu (Q_{0}-R_{0}\theta_{0}+E)$$

where T is an identity matrix that can be conformed. The behaviors of a linear system are highly dependent on the choice of  μ, the parameter, as well as the distribution of the eigenvalues of $T-\mu R_{0}$.

## 4. Experimental Results

This section outlines the experimental results of the proposed lightweight-guided capsule network. Each phase of the proposed model is evaluated using qualitative and procedural methods. The performance metrics [91], such as accuracy, recall, sensitivity, specificity, and AUC-ROC, were used to evaluate the effectiveness of this study’s skin lesion classification model. In this study, all the experimental tests were implemented using IDLE Shell 3.11.4 on a 4 GHz Intel Core i7 CPU at a rate of @ 1.80 GHz, 2304 Mhz, 4 Core(s), 8 Logical Processor(s), 12 GB of NVIDIA K80 GPU RAM, and 4.1 TFLOPS of performance. NVIDIA K80 GPU RAM is a powerful hardware accelerator that helps accelerate matrix multiplications and convolutions.

### 4.1. Dataset

Two datasets, namely, HAM10000 and ISIC2020, are considered in this study, acquired from the ISIC repository. All the images are labeled benign and malignant. The HAM10000 [92] dataset contains 10,015 dermoscopy images from patients in Australia and Austria, relevant patient history information and dermatologist annotations. The ISIC 2020 [93] dataset comprises 33,126 dermoscopy images of various benign and malignant skin diseases from over 2056 patients. It is observed that a limited number of images have annotations and binary masks, while most have clinical specifications.

Furthermore, the images are in JPG format, without pixel resolution data. Consequently, there is a shortage of information regarding the precise dimensions of the lesions, impeding the classifier’s training. The task of accurately classifying datasets becomes increasingly difficult due to the presence of images with various resolutions and imbalances in class distribution.

We evaluated the class imbalance [94] using ImbR (imbalance ratio), IntraC (intra-class distance), InterC (inter-class distance), DistR (distance metric), and Silho (silhouette score) on both datasets, as shown in Table 2. The intra-class (IntraC) and inter-class (InterC) metrics show the average distances between images in distinct and same classes. Image vectors were used to calculate both measures using Euclidean distance. The ratio (DistR) between these measurements showed similar distances, indicating substantial class overlap. Finally, the silhouette score (Silho) showed how similar each image is to its group relative to others. The results indicated a lack of strong correspondence between images and their respective classes. Furthermore, it was observed that even samples from distinct groups exhibited proximity in the feature space. Therefore, utilizing these metrics is advantageous in selecting appropriate fine-tuning parameters and optimizers to attain the required outcomes in data analysis or machine learning endeavors [94].

In our experiment, we utilized the standard dataset splitting approach, where 70% of the data were allocated for training, 15% for validation, and 15% for testing. The split was conducted using randomness to ensure a fair and balanced distribution among the subsets. The test dataset was exclusively allocated for the final evaluation of the trained model. The model construction and hyperparameter tuning steps were conducted without utilizing any information from the test dataset to prevent data leaking and ensure an unbiased performance assessment. The test dataset was utilized solely once subsequent to the finalization of the model. By managing the dataset with care, we assure the credibility of our reported results and reduce the possibility of overfitting to the test set while developing the model.

### 4.2. Image Preprocessing

**Figure 6:**
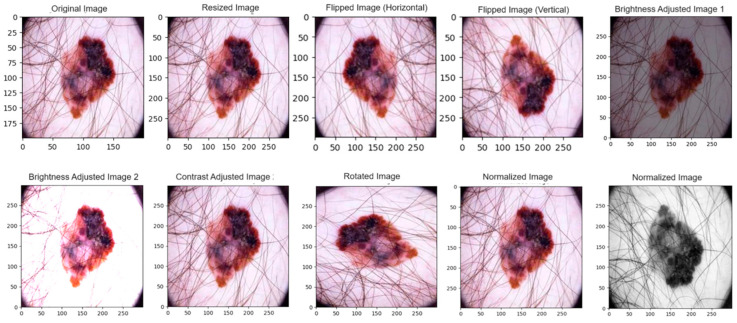
Augmented and normalized images.

The experimental results from the image processing phase are presented in this section. Table 2 shows the imbalanced datasets, which may compromise the network’s learning phase. To overcome this issue, synthetic images were generated for training from the class with the melanoma class’s fewest samples. Techniques such as data augmentation and normalization were employed to augment the size of the training dataset, resulting in the generation of processed data in the form of 299 × 299 JPG images. The input image was resized and normalized, then data augmentation was employed during the training phase to artificially increase the diversity of the training dataset and improve the model’s generalization capabilities. The augmentation techniques that were applied to the input images were (1) horizontal and vertical flip (2) brightness adjustment, (3) contrast adjustment and (4) rotation, as shown in Figure 6. To assess the influence of data augmentation on model performance, experiments were conducted without the application of any data augmentation techniques during the training phase. The model was trained and evaluated using the original, non-augmented dataset, as well as with the augmented dataset which is discussed in Section 4.6.

**Figure 7:**
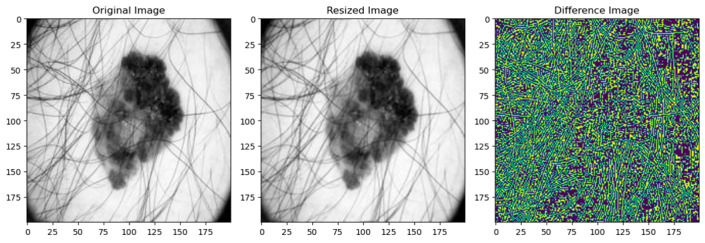
Absolute difference between original and resized image.

**Figure 8:**
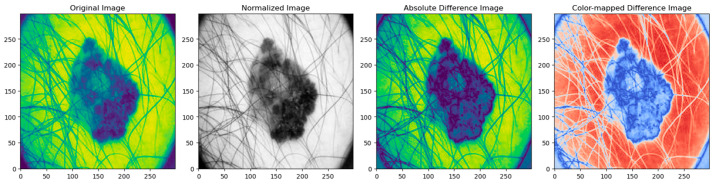
Absolute difference between original and normalized Image.

The quality of the image is evaluated using mean squared error (MSE), structural similarity index measure (SSIM), and peak signal-to-noise ratio (PSNR) [93] for resized and normalized images. Figure 7 illustrates the absolute pixel-wise difference between the original and resized images, which indicates how much the pixel values differ in the image. Brighter portions in the difference image show greater difference, whereas darker regions show more similarity. A white or brightly colored difference image indicates significant variations between the original and scaled photos, while a black difference image suggests identicality. The difference image shows where the images changed during resizing. This information helps comprehend the resizing operation and identify places that were affected more. The resized image is then normalized by scaling its pixel values to a standard range of [0, 1] or [−1, 1]. Figure 8 shows the absolute pixel-wise difference between the original and normalized images, indicating that normalization improves the convergence of the optimized algorithm’s overall dissimilarity between the images and quantifies the extent of image transformation during normalization. Table 3 shows the performance metrics in the preprocessing phase using PSNR, SSIM, MSE and mean absolute difference.

### 4.3. Active Contour Segmentation

**Figure 9:**
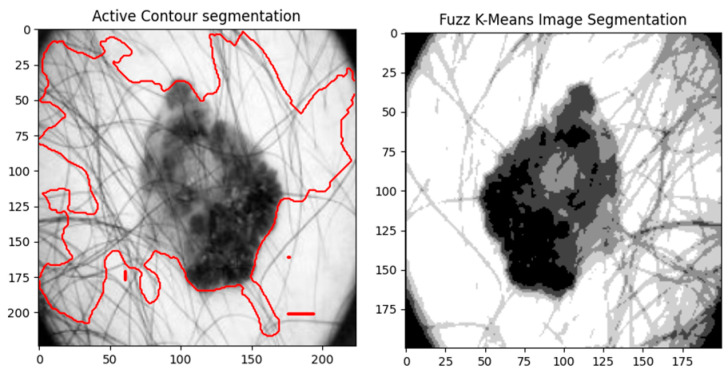
Comparison between active contour and fuzzy k-means.

The normalized image is fed into the active contour snake model to remove irrelevant information from the image. This model uses a deformable curve to capture the object boundaries of the image. It is used to define smooth shapes in images, build closed contours for regions, and find irregular shapes in images. We compared AC with fuzzy K-means image segmentation, as shown in Figure 9. It shows that the borders between various regions in the skin lesion images are not well delineated and exact by using FKM.

**Figure 10:**
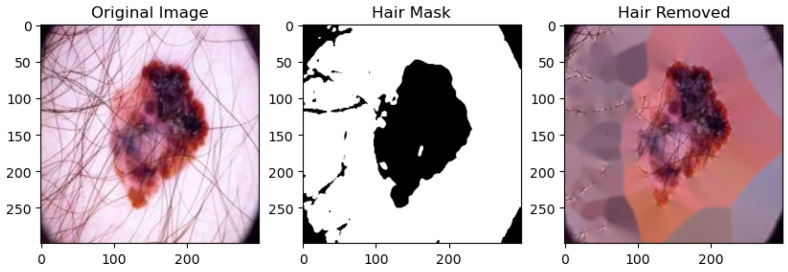
Hair mask and removal.

After applying active contour, hair detection and inpainting techniques are used to fill the hair region, as shown in Figure 10.

### 4.4. Feature Extraction

**Figure 11:**
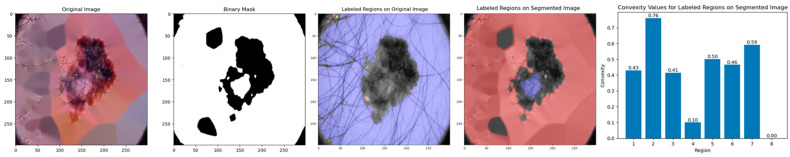
Convexity values for labeled region.

**Figure 12:**
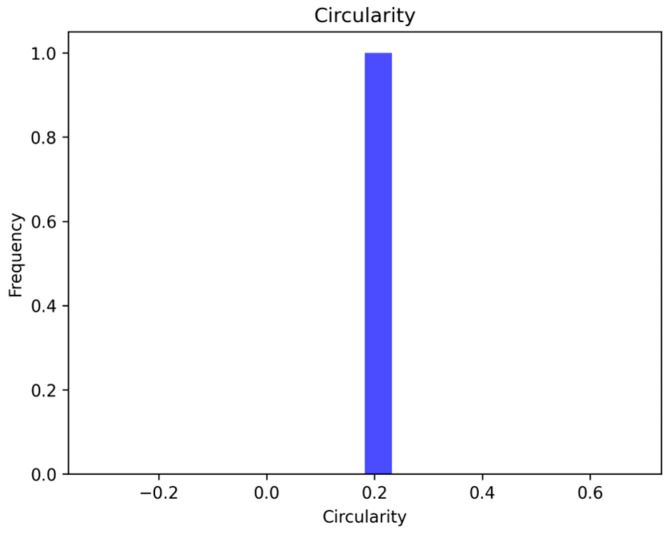
Circularity values for labeled region.

**Figure 13:**
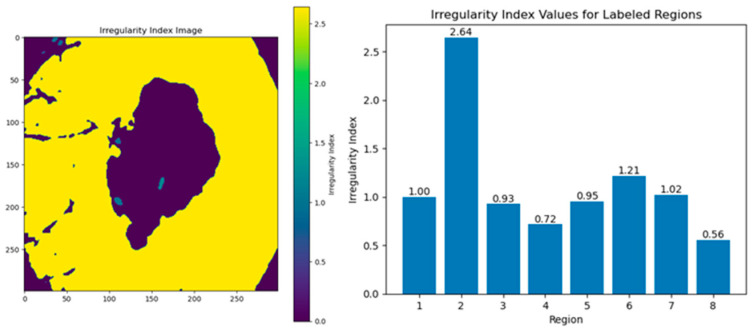
Irregularity index values for labeled regions.

**Figure 14:**
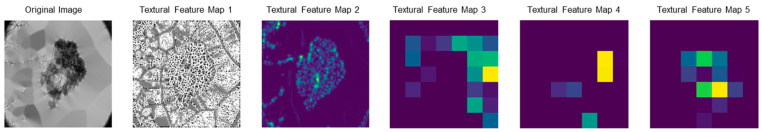
Texture pattern map.

**Figure 15:**
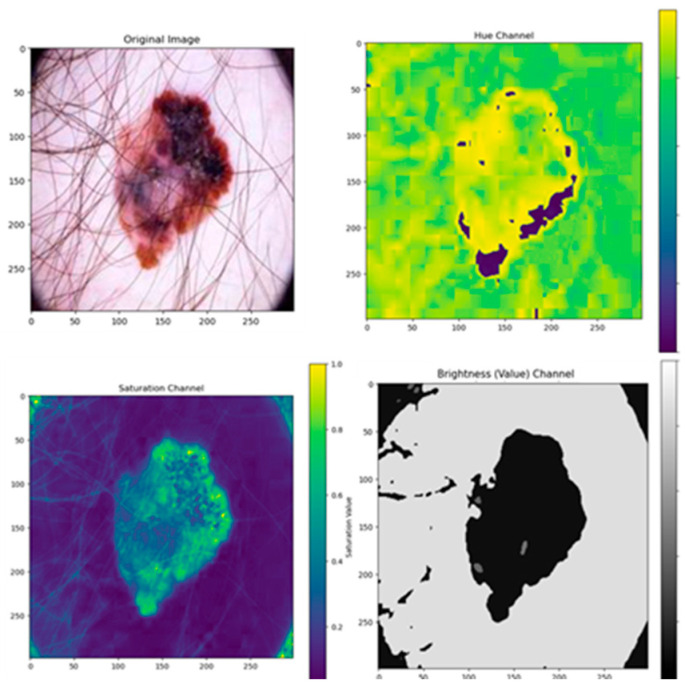
Color feature representation.

**Figure 16:**
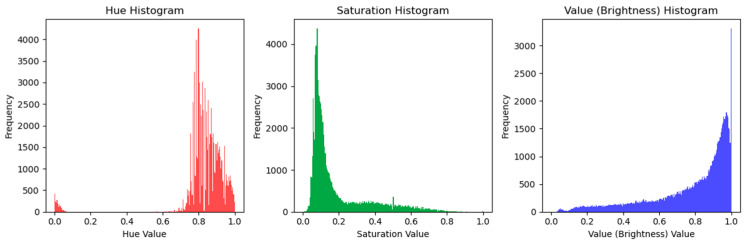
Color feature histogram representation.

The ResNet50 transfer learning algorithm was employed as a feature extractor in detecting skin cancer. Using pre-trained deep learning knowledge enables the effective representation of skin image data by implementing a robust approach. The retrieved features encompass a range of characteristics such as convexity, circularity, irregularity index, textural patterns, color features, and region of interest, as shown in Figure 11, Figure 12, Figure 13, Figure 14, Figure 15 and Figure 16. The utilization of this technique has the potential to significantly augment the effectiveness and efficiency of algorithms employed in the identification of skin cancer.

#### 4.4.1. Convexity

The convexity of the image is computed to quantify the extent to which a region of interest is convex, as it measures the ratio between the area of interest and the location of its convex structure. To assess the convexity of the image, a Gaussian smoothing technique is employed on the grayscale image, creating a binary mask. The range of the convexity value is between 0 and 1, with a value of 1 indicating a perfectly convex shape. The convexity for each labeled region is computed using the contour, as shown in Figure 11. The convexity value for region 2 is 0.76, indicating that the region has a relatively high degree of convexity and is close to a regular shape.

#### 4.4.2. Circularity

Circularity is a geometric attribute to quantify the circular nature of the region, and the values range between 0 and 1. Figure 12 shows that the area-to-perimeter ratio matches the characteristics of the circular region.

#### 4.4.3. Irregularity Index

The irregularity index is used to quantify the irregularity of the region’s shape in the image. It is one of the most critical lesion features in predicting malignancy. Borders exhibiting an irregularity index exceeding 1.8 were categorized as irregular. Because of the significant difference in incidence between benign and malignant skin lesions, accurate assessment of irregular boundaries is clinically crucial. As shown in Figure 13, region 2 has an irregular index.

#### 4.4.4. Textural Pattern

Texture is a characteristic utilized to divide and classify regions of interest within images. Texture imparts insight into the spatial configuration of hues or levels of intensity within a given image, as shown in Figure 14.

#### 4.4.5. Color Features

Color features represent the image’s color characteristics by defining the color space feature appearance, such as hue, saturation, and brightness, as shown in Figure 15, and the histogram of these color features is depicted in Figure 16.

### 4.5. Results of Classification Phase Using Proposed Lightweight-Guided CapsNet Model

The feature maps obtained from the convolution block of ResNet50 are then fed into CBAM to generate the corresponding channel and spatial attention map. The attention map suppresses the irrelevant areas and highlights the critical regions of the feature map. The resultant features are inputs to the guided CapsNet model to predict the lesion. To evaluate the proposed model’s ability to apply new or unfamiliar data, accuracy, sensitivity, specificity, AUC-ROC, recall, and F1 score are used to measure how effectively the model can generalize to such data. We used attention guidance fusion to improve the model’s parameters during the classification phase. SGDM optimizer is used to optimize the model. To improve the model’s performance accuracy and computational efficiency, 0.36 G FLOPs is applied to the proposed model. Table 4 depicts the classification accuracy, number of parameters and FLOPs on the test dataset. It is worth noting that LA-CapsNet has 0.36 billion FLOPs, potentially reducing its computational requirements by up to 10 times or more. It makes the model more versatile and applicable to various devices and applications, particularly in resource-constrained environments like mobile devices or embedded systems.

**Figure 17:**
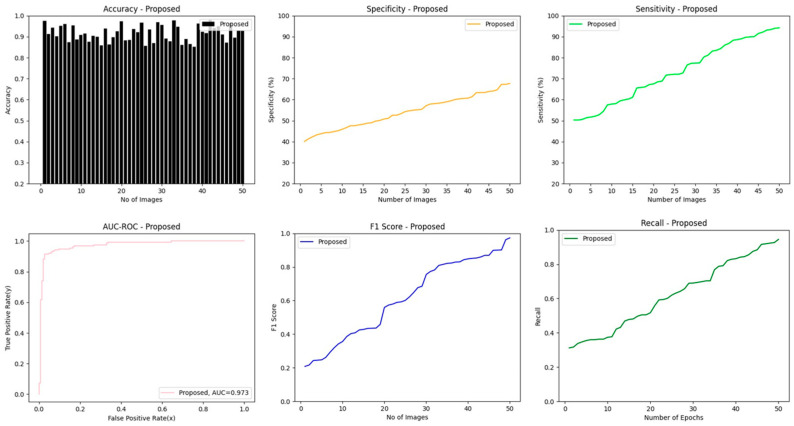
Performance evaluation of the proposed model.

**Figure 18:**
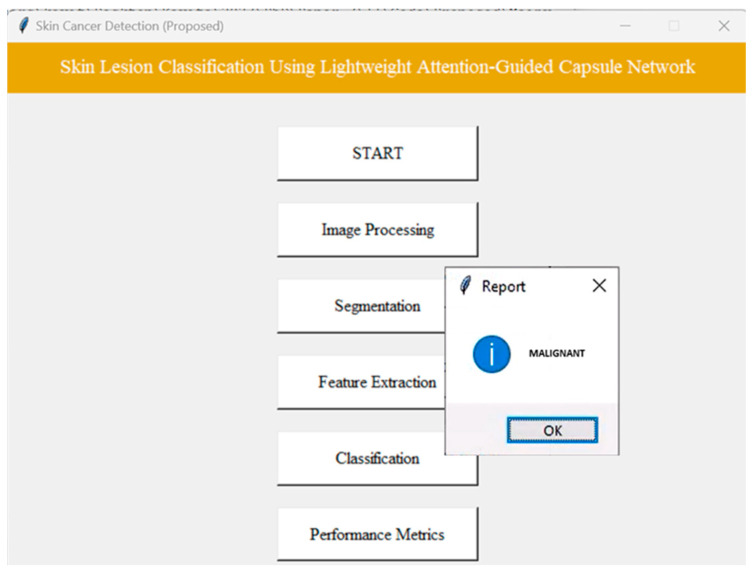
GUI interface.

Figure 17 shows the performance of the proposed lightweight-guided CapsNet model. The model achieves an accuracy of 98.04%, specificity of 68%, sensitivity of 96%, AUC-ROC of 97.3%, F1 score of 99% and recall of 98%. We employed a GUI interface to navigate each phase of the proposed model execution, as shown in Figure 18.

### 4.6. Comparison of LACapsNet Results with Augmentation and without Augmentation

In this section, we present a comparative analysis of the performance of the LA-CapsNet model under two different conditions: with data augmentation and without data augmentation, as shown in Table 5.

The results obtained without data augmentation are used as a baseline to highlight the significance and benefits of employing augmentation techniques. Employing data augmentation demonstrates a positive impact on the model’s performance, robustness, and generalization capabilities.

### 4.7. Comparative Analysis

The proposed lightweight-guided capsule network was compared to current state-of-the-art methods, such as extreme learning machine learning-based optimization (ELM-TLBO), R-CNN, deep convolution neural network (DCNN), differential evolution artificial neural network (DE-ANN), and fuzzy k-means models. The following performance indicators were compared using ISIC2020 and HAM10000 datasets: accuracy, sensitivity, specificity, recall, AUC-ROC, and F1 score.

#### 4.7.1. Accuracy

The dataset is analyzed to determine the percentage of accurately classified instances in cases of skin cancer:

(27) $$A_{C}=\frac{O}{N}$$

where N (total number of predictions) shows the total number of skin cancer instances for which the model has produced a forecast, and O (number of correct predictions) represents the total number of cases of skin cancer accurately represented by the model.

**Figure 19:**
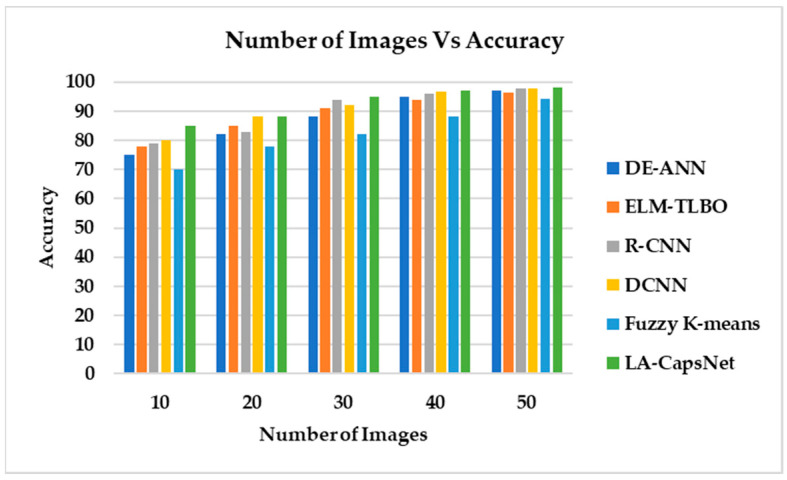
Number of images vs. accuracy.

Figure 19 illustrates the classification accuracy as the number of images increases. It is worth noting that the proposed model exhibits a higher detection accuracy compared to other SOTA methods. A detection accuracy of 97.83% is attained by DCNN, 96.97% by DE-ANN, 96.21% by ELM and 98.04 by the proposed LA-CapsNet for 50 images. Compared to existing approaches, our proposed method exhibits higher accuracy.

#### 4.7.2. Sensitivity

When utilizing deep learning for skin cancer detection, sensitivity is referred to as true-positive rate T, which assesses how well the model can identify positive cases or, more precisely, how well it can identify malignant skin lesions. The matrices equation follows as:

(28) $$S=\frac{T}{T+f}$$

where T(True positives) represents the number of skin cancers that were correctly identified as positive and $f\left(False\;Negatives\right)$ denotes the number of actual skin cancers that the model mistakenly dismissed as being free of the disease.

**Figure 20:**
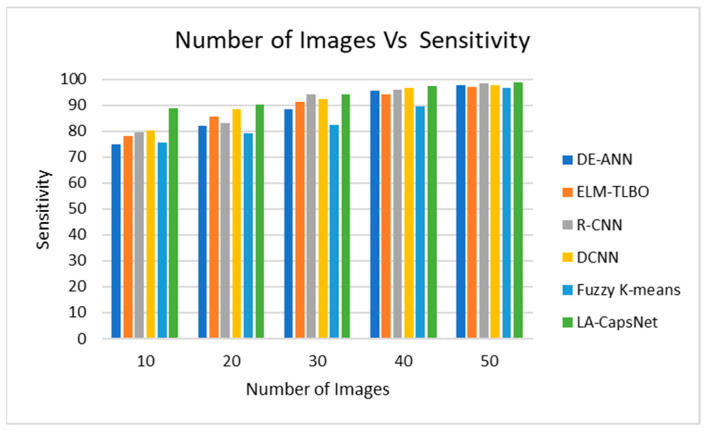
Number of images vs. sensitivity.

The comparison of simulation results using current and proposed methodologies in the environment of skin cancer detection reveals a notable trend: sensitivity increases as the number of images grows, as depicted in Figure 20. Its heightened sensitivity can be attributed to the enhancements in image quality and processing capabilities introduced by our proposed approach to sensitivity. Current methods include DE-ANN and DCNN. When the images reached their maximum of 50, the proposed work was 98.82%; however, the existing results achieved 85% and 90%, respectively.

#### 4.7.3. Specificity

Specificity quantifies the proportion of healthy skin instances correctly categorized as negative, essential for providing patient comfort and avoiding unnecessary treatments. It evaluates the model’s capacity to minimize false alarms by determining how many healthy skin cases are correctly classified as negative.

(29) $$SP=\frac{C}{C+P}$$

where C (true negatives) reflect the number of cases accurately recognized as negative and P (false positives) indicate the number of cases that should have been classed as negative but were instead misclassified as positive.

**Figure 21:**
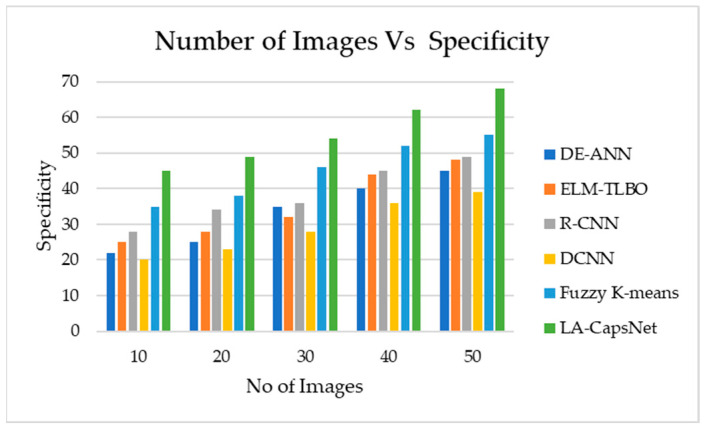
Number of images vs. specificity.

The proposed method yields the maximum feasible specificity to assess efficient specificity. Figure 21 illustrates how specificity declines with increasing images, yet the suggested method achieves higher specificity than other methods like DCNN and DE-ANN. The specificity of the proposed approaches is 49 in 25 images and 68 in 50 images. In 25 photographs, DCNN will obtain a specificity of 39, and in 50 images a specificity of 48. After 25 images, DE-ANN will achieve a specificity of 28 after 50 images and a specificity of 37.

#### 4.7.4. AUC-ROC

An increased AUC-ROC score signifies an enhanced capacity of the model to generate precise forecasts, an essential attribute for prompt identification and assessment of skin cancer.

(30) $$R=\frac{T}{T+P}$$

The number of accurately identified positive cases is represented by T (true-positive rate), and P stands for false positives—positive cases mistakenly considered negative.

**Figure 22:**
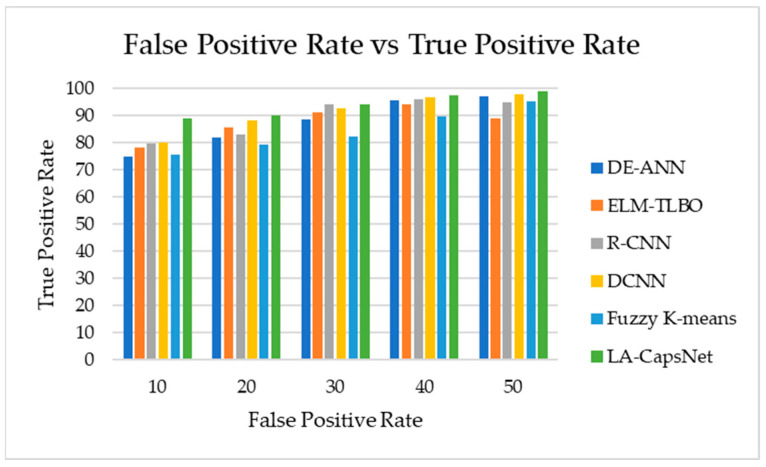
False-positive rate vs. true-positive rate.

Figure 22 shows the true-positive rate with the false-positive rate. The proposed model has a high true-positive rate compared to other approaches, such as ELM-TLBO and R-CNN. The proposed methods have a maximum true-positive rate of 99%, R-CNN achieved a maximum true positive rate of 95%, and ELM-TLBO achieved a maximum true-positive rate of 89. Compared to other approaches already in use, the one we have developed is more precise.

#### 4.7.5. F1 Score

We proposed a method that reasonably evaluates a classifier’s performance in merging recall and precision obsessed by a solitary score. The F1 score is computed as:

(31) $$F_{s}=\frac{2\times T\times R_{C}}{T+R_{C}}\;$$

The ratio of genuine positives to all cases categorized as positive is known as precision (W). The true-positive rate is recall (K).

**Figure 23:**
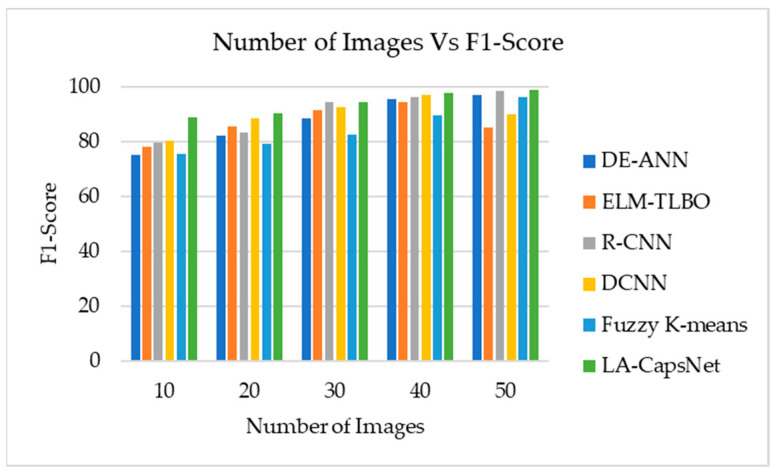
Number of images vs. F1 score.

Figure 23 shows that the proposed method outperforms current approaches like fuzzy k-means and ELM-TLBO regarding F1 score. The suggested methods’ F1 score is 85% in 30 images and 98.87% in 50 images. DCNN achieved an F1 score of 73% in 30 images and an F1 score of 90% in 50 images. ELM-TLBO will obtain an F1 score of 65% after 30 images and an F1 score of 85% after 50 images.

### 4.8. Research Summary

The main objective of the proposed model is to employ lightweight-guided CapsNet model algorithms to diagnose skin cancer. First, the input images are loaded with ISIC 2020 and the HAM10000 dataset. The next preprocessing step involves adjusting the raw image by enhancing, scaling, and normalizing it. Next, we proceed with the segmentation procedure. For this stage, we will use the active contour segmentation technique. We extract properties such as convexity, circularity, irregularity index, textural patterns, color attributes, region of interest, etc., using the ResNet50 transfer learning algorithm during the feature extraction stage. After that, we proceed with the classification. We optimized the model’s parameters during this phase using attention guidance fusion. Accuracy, sensitivity, specificity, AUC-ROC, recall, and F1 score are the accomplishment metrics used to validate the proposed technique. As evident from Table 6, LA-CapsNet exhibits superior performance across all metrics, demonstrating its effectiveness in skin lesion classification. Its ability to capture local and global features and its robustness with varying image quality make it a promising approach for clinical applications.

## 5. Conclusions and Future Scope

This study introduced a lightweight-guided capsule network called LA-CapsNet for skin lesion classification by fusing attention mechanisms. The HAM10000 and the ISIC 2020 datasets were used for early skin cancer diagnosis in this investigation. The datasets were preprocessed using resize, augmentation, and normalization. Segmentation is achieved using the active contour snake model. The RESNET50 model uses such features as convexity, circularity, irregularity index, textural patterns, and color. Normal, benign, and malignant classification utilizes the CapsNet and optimization (stochastic gradient descent) models. The proposed lightweight-guided CapsNet achieved an accuracy of 98%, Sensitivity of 98.82%, Specificity of 68%, AUC-ROC of 99%, and F1 score of 98.87%. According to the numerical analysis, our solution performs better than all other methods currently used in every metric. Although capsule activations offer some understanding of the model’s decision-making process, they are less easily understood than feature maps in convolutional neural networks. This can create difficulties in comprehending the process by which the model formulates its predictions. In future, we aim to investigate explainable AI (XAI) techniques to gain an understanding of the LA-CapsNet decision-making process.

## Figures and Tables

**Table 1:** Summary of literature review.

Ref.	Objective	Methods/Techniques	Research Gap
[45]	Deep learning-based skin cancer detection using dermoscopy images.	Deep neural network algorithms such as faster R-CNN and fuzzy k-means clustering (FKM)	When employing FKM, the boundaries between distinct areas in the skin lesion images cannot always be clear and precise.
[46]	Techniques for detecting skin cancer that categorize the disease as benign, malignant, or normal.	Fuzzy C-means clustering (FCM), rider optimization algorithm (ROA)	FCM clustering faces challenges in complex or textured images, leading to weak convergence and local minima issues, impacting image segmentation quality.
[47]	To categorize dermoscopy images into benign or malignant lesions.	CNN, support vector machines (SVMs)	The proposed system does not emphasize preprocessing. Thus, it affects input image accuracy.
[48]	To improve dermoscopy image learning and skin cancer diagnosis training.	DNN, DL models	DNNs require a lot of labeled data for training, making it hard to find and annotate diverse and accurate skin lesion images, especially for rare or specialized malignancies.
[49]	Deep learning-based melanoma classification.	CNN, super-resolution generative adversarial networks (SRGANs)	CNN may make decision-making and learning features challenging to interpret. The final prediction is complex, with more extracted features.
[50]	Skin cancer detection using ML and image processing	Image super-resolution (ISR) algorithms	ISR image artifacts can affect skin cancer detection. Abnormalities lead to generating diagnostic false positives and negatives.
[51]	Teaching–learning-based optimization for detecting skin cancer.	TLBO algorithm, extreme learning machine (ELM)	The suggested technique requires a lot of computing power to handle large skin cancer imaging datasets, limiting its practical uses.
[52]	An explainable CNN-based method for early melanoma skin cancer detection.	CNN-based stacked ensemble architecture	Stacking ensemble frameworks with many models, such as CNNs, can create a complex architecture. Complexity needs more extended training and more resources.
[53]	Detecting skin cancer using transfer learning	MobileNetV2	Due to its low capabilities, MobileNetV2 can have difficulty with complex skin diseases that demand fine-grained characteristics.
[54]	Deep learning-based skin cancer detection and categorization.	Swallow swarm optimization (SSO), DLCAL-SLDC method, CAD model	When CAD systems overlook carcinogenic lesions and misclassify benign lesions as malignant, false positives and negatives occur. Errors cause needless biopsies or missed diagnoses.
[55]	DL-based skin cancer classifier for imbalanced datasets.	Modeling based on deep learning RegNetY-320, InceptionV3, and AlexNet	Most of these parametric algorithms require uniform data, but without controlling their nature. Thus, these approaches cannot accurately diagnose the condition.
[56]	Network of capsules for skin cancer diagnosis	FixCaps, convolutional block attention module	FixCaps’s generalization performance has not been thoroughly investigated.
[57]	Detect skin cancer from food antioxidants via deep learning.	CNN, DL model	The suggested system for effective training considers features, classifications, and augmentations, which can overfit data.
[58]	A robust skin cancer detection system using transfer learning.	Optimizing particle swarms (PSO) with dynamic-opposite learning	Proper transfer learning depends on the quantity and quality of the target skin cancer dataset. Transfer learning fails if the dataset is too small or has noisy or biased samples.
[59]	DL approaches for detecting and categorizing skin cancer	CNN, medical vision transformer	Privacy considerations and the rarity of some skin cancers make obtaining datasets for skin cancer detection and expert annotations difficult.
[60]	DL model- based classification for skin cancer	CNN, EfficientNet-B0, ResNet-152, Vgg-16, Vgg-19, Inception-V3, and MobileNet	DSCC_Net model works only for light-skinned people. This study omitted dark-skinned people.
[61]	Convolutional neural network for cancer classification	CNN, Grad-CAM	Due to computational costs, access to strong GPUs or cloud computing resources is necessary to train optimized CNN designs.
[62]	Skin cancer classification via medical vision	Medical vision transformer (MVT), multilayer perceptron (MLP)	MLPs do not capture image spatial connections. Skin cancer diagnosis often requires spatial patterns and specific features.
[63]	Melanoma identification from dermoscopy images using DL	GrabCut-stacked convolutional neural networks (GC-SCNNs), SVM	GrabCut can encounter issues with complex backdrops or parts with similar color distributions to the target object. The algorithm cannot distinguish foreground from background in some cases.
[64]	Skin cancer detection model based on feature fusion	Local binary patterns (LBPs), LSTM	LSTM is commonly used for sequential data, including time series or natural language word sequences. This method can convert images into sequential representations, although it cannot be as efficient or precise as convolutional neural networks.

**Table 2:** Skin lesion dataset-class assessment metrics.

Class Assessment Metrics Using Randomly Sampled Datasets						
Dataset	No. of Images	ImbR	IntraC	InterC	DistR	Silho
HAM10000 [92]	7818	6.024	8705	9770	0.891	0.213
ISIC 2020 [93]	25,838	9.012	28,786	32,132	0.804	0.202

**Table 3:** Performance of image after preprocessing phase.

	PSNR (dB)	SSIM	MSE	Mean Absolute Difference
Resized image	33.52	0.97	0.0023	109.01
Normalized image	44.90	0.97	0.0023	0.0052

**Table 4:** Classification accuracy (%) on ISIC2020 test data.

Model	Accuracy (%)	Params Millions (M)	FLOPs Giga-Billions(G)
DE-ANN	96.97	4.3	1.2
ELM-TLBO	96.21	4.3	1.2
R-CNN	97.63	1.2	1.2
DCNN	97.83	1.2	1.2
Fuzzy K-means	94.23	1.2	1.2
**LA-CapsNet**	98.04	1.2	0.36

**Table 5:** Comparison of key performance metrics with and without data augmentation.

LA-CapsNet	Accuracy	Sensitivity	F1 Score	AUC	Specificity
With data augmentation	98.04	98.82	98.87	99.00	68.00
Without data augmentation	78.00	81.04	78.84	78.02	55.20

**Table 6:** Comparison of proposed model with SOTA methods.

Performance Metric	DE-ANN	ELM-TLBO	R-CNN	DCNN	Fuzzy K-Means	LA-CapsNet
Accuracy	96.97	96.21	97.63	97.83	94.23	98.04
Sensitivity	97.91	97.03	98.32	97.72	96.53	98.82
F1 score	97.01	85.00	98.42	90.00	96.07	98.87
AUC	97.21	89.00	95.00	97.92	95.43	99.00
Specificity	45.00	48.00	49.00	39.00	55.00	68.00

## Data Availability

The experimental datasets used to support this study are publicly available on data repositories at https://challenge2020.isic-archive.com (accessed on 23 October 2023).
